# Why do many pheasants released in the UK die, and how can we best reduce their natural mortality?

**DOI:** 10.1007/s10344-018-1199-5

**Published:** 2018-06-22

**Authors:** Joah R. Madden, Andrew Hall, Mark A. Whiteside

**Affiliations:** Centre for Research in Animal Behaviour, Psychology, University of Exeter, Exeter, EX4 4QG UK

**Keywords:** *Phasianus colchicus*, Game shooting, Non-native, Artificial rearing, Mortality

## Abstract

Around 60% of pheasants released for shooting in the UK, an estimated 21 million birds, do not end up at their intended fate: being shot. This constitutes wastage, raising economic, environmental and ethical questions. We review what is known of the fates of released pheasants and consider why they do not directly contribute to the numbers harvested. We focus on four main explanations: predation, disease, starvation and dispersal, and highlight other important causes of mortality. For each explanation, we attempt to attribute levels of loss and identify timings or conditions when such losses may be heaviest. We review factors that exacerbate losses and methods available to mitigate them. Opportunities for amelioration may arise at all stages of the rearing and release of pheasants and involve changes to the conditions under which eggs are produced, the way young pheasants are reared or the management of the environment into which they are released. We found few studies investigating impacts of post-release management techniques on pheasant survival outside of the breeding season within a UK context. We found that a number of less commonly deployed practices focusing on early-life, pre-release management may improve survival. Given the scale of pheasant releasing in the UK, even improvements in survival of 1% would mean that ~ 350,000 fewer birds die of natural causes. Complementing current post-release management with proven novel pre-release management interventions could reduce the number of pheasants required for release, whilst maintaining current shooting levels. Lowering release numbers would lower financial costs, benefit the environment and reduce some ethical concerns over the release and shooting of reared pheasants.

**Electronic supplementary material:**

The online version of this article (10.1007/s10344-018-1199-5) contains supplementary material, which is available to authorized users.

## Introduction

Each year, pheasants *Phasianus colchicus* are artificially reared in captivity (hereafter ‘reared’) and released into the UK countryside for game shooting. Numbers of released game birds have been put at 25 million (Sage et al. 2005), 34.9 million (BASC 2015), 40 million (PACEC 2008) and 50 million (including partridges at likely < 20%) (Harper 2014; Winter 2013). Interpretations of such data vary and exact numbers are disputed. Regardless, there is a general pattern of increased release numbers over the past 50 years, with around nine times as many pheasants released in 2011 compared to 1961 when monitoring began (Robertson et al. 2017). Consequently, they now comprise around 23% of the mass of the UK bird breeding population (Eaton et al. 2012) and hence constitute a potentially influential component of the British ecosystem (Mustin et al. 2018, Roos et al. 2018).

The numbers of birds being shot have not increased to the same extent. Currently, there is no legal requirement to record the numbers of birds being reared or shot, but crude bag counts commonly put return ratios at 35–40% of the total number of birds released (Robertson et al. 2017). When considering only pheasants released at one site and shot on the same estate (confirmed by including only tagged birds), return rates are lower with 28.3% of 20,950 pheasants at six sites over 3 years (Turner 2007) and 20.4% of 26,502 pheasants at eight (different) sites over 6 years (Madden, unpublished data from shoots releasing > 600 birds). This disparity between crude and tag counts may be accounted for by immigration and emigration from and to neighbouring shooting estates, misattribution because of tag loss or supplementation from wild populations.

Assuming an annual release of 35 million pheasants and a return rate of 40%, around 21 million released pheasants are not harvested and so cannot benefit the shooting industry. This has economic, environmental and ethical implications. Each pheasant costs £12.86 to rear and release (Anonymous 2016); therefore, the 21 million pheasants that do not contribute to the annual harvest constitute an economic loss of £270 million per year. Although release of game birds and their subsequent shooting can stimulate significant environmental management that benefits a wider range of species and habitats (Mustin et al. 2018), release of game birds, especially at high numbers/densities, can also contribute to environmental damage. This includes adverse modification of woodland ground flora and fauna within their immediate release pens (Neumann et al. 2015; Sage et al. 2005) and impacts on the broader environment (Callegari et al. 2014; Sage et al. 2009). Finally, the waste of so many birds also raises ethical questions, especially when eggs are produced and young birds reared in unnatural commercial conditions before being released to face natural perils such as starvation or predation (Matheson et al. 2015). Therefore, it is imperative to understand why pheasants that are reared and released in the UK explicitly to shoot do not meet their intended fate.

We surveyed the academic and grey literature to determine levels and timings of mortality for wild and released pheasants, with an initial focus on data from the UK, based on queries of Google Scholar and Web of Science (search terms: “pheasant” & “mortality” or “survival”). We then followed up references from this first set of papers. In addition, we consulted with researchers at the Game and Wildlife Conservation Trust who provided access to additional unpublished reports and theses. Relatively little work has been published on pheasant ecology in the UK since the 1980s, so we also draw on research from Europe and the USA to provide figures for particular causes of mortality, but substantial differences in both game management techniques and hunting styles between the regions mean that direct comparisons must be made with caution (see ESM for descriptions of regional differences).

## The losses of released and wild pheasants

**Fig. 1:**
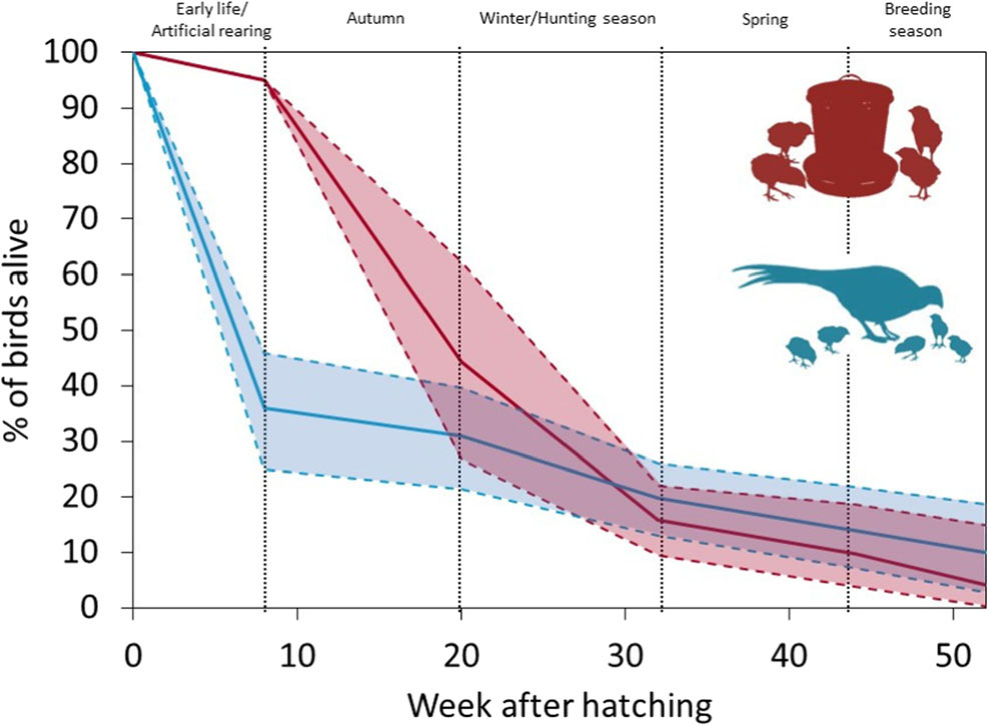
Projected mean survival of reared (red) and wild (blue) pheasants from hatching for 1 year. Due to the paucity of information, we reviewed literature covering survival of reared and wild pheasants from both the UK and worldwide. From this literature, we extracted mean (solid lines) and max/min (dashed lines) survival rates for birds at each stage of their first year of life. Data from which survival estimates were calculated is presented in the ESM Table 1

The loss of reared pheasants starts from the day of their release into the wild (Fig. 1). This may involve terminal loss from mortality or functional loss from dispersal away from the estate/farm where they were released, such that they are not available to be harvested by the persons who released them. Mortality of reared birds in the first 6 weeks of life prior to release is typically uniform at < 5% (Đorđević et al. 2010) and significantly less than the mortality rate of wild-born broods over the same period (between 12 and 100% mortality (Hill and Robertson 1988b)). Losses of reared birds are typically heaviest in the first few weeks post release, a rate that decreases with time (Fig. 1). In the UK, over 3 years across six sites, 42% of reared pheasants, for whom fate was known, were dead of natural, non-shooting, causes before the end of the first shooting season in February. A further 45% were shot (both on and off the estates where they were released), leaving just 13% still alive at the end of the season (Turner 2007). Crude adjustment suggests that natural mortality (excluding shooting) from release to the start of February runs at 61%. In the relatively few releases of reared birds in the USA, where management such as predator control is rare, mortality may reach levels of 60–85% in the first 2 months post release (Burger 1964; Hessler et al. 1970; Krauss et al. 1987). These mortality levels compare poorly to those of 2-month-old wild-born birds that have survived to a similar period (September–April) in the USA, which range from 43 to 48% (Clark et al. 2008).

After the shooting season, survival varies across sites and years and may differ with sex and origin. Reared birds in the UK had a 41% chance of surviving February–May, compared to 65% for wild-born birds at the same site (Hill and Robertson 1988a). At another site, birds had an 84% chance of surviving February–April, with a 24% chance of surviving February–August (Hoodless et al. 1999). Consequently, only a small proportion of released birds (likely ~ 9% Hoodless et al. 1999) survive to the start of the breeding season. During the breeding season, released females have lower incubation success (Sage et al. 2003) and sometimes lose the condition while incubating, compared to wild females, such that they cannot fly or even move from their nests (Robertson 1997). Survival of hens during the breeding season has been well studied in the USA, with survival of reared hens (4% in 2000, 8% in 2001) being lower than their wild-born conspecifics (40% in 2000 and 43% in 2001) (Musil and Connelly 2009). The consequence of these compounding mortality rates is that a very small percentage of the released population is likely to survive to the shooting season in the following year. Our own data (Madden and Whiteside, unpublished data) reveals that across two sites, of 2652 tagged birds released, only 42 (1.6%) were shot in the following year, and Turner (2007) reports that < 1% of 20,950 tagged birds were shot in the following years. Therefore, reared pheasants only really contribute to the harvest in the year that they were released, suggesting that the majority of reared pheasants released in the UK are dead within 15 months of release.

## Opportunities for game managers to reduce mortality

These patterns of mortality prompt us to identify what factors are causing the high losses post-release and review methods to ameliorate them. Game managers can intervene at several points in the rearing and release process (summarised in Table 1). We provide a more detailed description of normal practice for the rearing and release of pheasants in the UK in the ESM to assist readers unfamiliar with the process.

**Table 1:** Interventions thought to improve the survival of released pheasants, along with causes of mortality that they are likely to ameliorate

Stage of intervention	Intervention	Likely effects	Evidence for increased survival/decrease mortality?	Presumed to reduce mortality due to:			
				Predation	Disease	Starvation	Dispersal
Egg production	Use of eggs from wild birds; alter diet of laying hens	Reduces stress during rearing^1^; Improves learning ability^3^			✓	✓	
Early life rearing	Rearing with surrogate parents	No effect on dispersal distance^26^					
	Provision of elevated perches	Promotes roosting off the ground^7,8^; enhances morphology for perching^7,8^; improves spatial memory^7^; reduces stress^7,8^	Yes^7^	✓	✓		✓
	Provision of enriched rearing environment	Reduces stress^2^; improved flight muscle development^6^		✓	✓		✗ (improved flight may make dispersal easier)
	Anti-predator training (red-leg and Chukar partridge, but not tried for pheasants)	Improved vigilance	(Yes)	✓			
	Provision of diverse diet including live prey	Increases foraging efficiency^9^; alters gut morphology^9^; assists transition to natural diet^9,23^	Yes^9^	✓		✓	✗ (may encourage dispersal to search for alternative food supplies)
	Vitamin E supplement	Reduces parasite load and oxidative stress in later life^19^			✓		
Point of release	Reduced stocking density	Reduces risk of disease transfer^13,14,15,16^; decreases area outside release pen utilised by males^25^; decreases susceptibility to coronavirus^19^; reduced attractiveness of area to predators^4,5^		✓	✓		✓
	Moving feeder sites and release pens regularly	Reduces density of gapeworm eggs in local area^21^			✓		
Post-release	Predator control	Reduces predator threat	Only at large scales ^10,11^	✓			
	Supplementary feeding	Increases food availability; can reduce distance pheasant must travel from cover to forage^12^; maintains body condition^23^	No (females)^22^	✓	✗ (Concentrated feeding may increase disease transfer risk)	✓	
	Anti-helminthic treatment (oral dosing or via feed)	Reduces worm load; reduced detectability on nest by predators^15^; increases chick production^20^	Yes^15^	✓	✓		
	Aracicide treatment (necklaces)	Reduces tick load^17,18^; improves chance of acquiring harem (males)^17^; improves hatching rate (females)^18^	Yes (females)^18^	✓	✓		
	Provision of suitable habitat	Reduces dispersal; avoid human disturbance/threats e.g. traffic^24^					

^1^(Santilli et al. 2004); ^2^(Hrabcakova et al. 2012); ^3^(Bagliacca et al. 2000); ^4^(Kenward et al. 1981); ^5^(Robertson 1988); ^6^(Robertson et al. 1993); ^7^(Whiteside et al. 2016); ^8^(Santilli and Bagliacca 2017); ^9^(Whiteside et al. 2015); ^10^(Frey et al. 2003); ^11^(Trautman et al. 1974); ^12^(Hoodless et al. 2001); ^13^(Draycott and Parish 2000); ^14^(Draycott and Parish 2000); ^15^(Draycott et al. 2006); ^16^(Gethings et al. 2015b); ^17^(Hoodless et al. 2002); ^18^(Pennycott 2000); ^19^(Orledge et al. 2012b); ^20^(Woodburn et al. 2002); ^21^(Gethings et al. 2015a); ^22^(Hoodless et al. 1999); ^23^(Draycott et al. 1998); ^24^(Bagliacca et al. 2010); ^25^(Turner 2007); ^26^(Ferretti et al. 2012)

Early life experiences can influence the development of behaviour, morphology, physiology and cognition across a range of taxa (Buchanan et al. 2013; Lindström 1999; West-Eberhard 2003). Having control over such an important developmental stage is crucial, as the lack of or provision of wrong stimuli can promote maladaptive characteristics. We believe that current pheasant rearing methods, prior to release, are tightly controlled by game managers, highly artificial and conducted in the absence of parents, in relatively barren environments at unnaturally high densities.

Game managers may assist pheasants post-release in several ways. They commonly supply feed at set points before and throughout the shooting season, with some continuing to feed after this (Draycott et al. 1998; Draycott et al. 2006; Hoodless et al. 2001). Managers can control predator numbers and may modify the landscape to provide favourable habitats to retain the birds in the shooting area during the shooting season and to provide nesting or feeding areas to support birds after the shooting has ceased.

## Causes of loss and mitigating interventions

### Predation

#### The effect of predation

Predation is the most common cause of mortality attributed to pheasants. Estimates of predation rates should be treated with some caution, as scavenging of remains can cause ambiguity as to the cause of death. However, we treat reports of pheasants found partially eaten or dismembered as having been killed by a predator. Across six UK shooting estates where predator control was implemented, 21% of reared birds whose fate was known were predated before the start of the shooting season, with a further 11% being predated or scavenged during the shooting season (Turner 2007). Most (~ 70%) recorded predation of reared pheasants in the UK is attributed to foxes (*Vulpes vulpes*) (Robertson 1988; Sage et al. 2001; Woodburn 1999). Raptors are implicated in < 1% of deaths of newly released pheasants, but on some sites, they are responsible for > 10% of deaths (Parrott 2015). Predation is especially common immediately after release. One release pen in Ireland suffered the highest rate of loss (48%) in the first 10 days after the birds left the release pen (Robertson 1988). Likewise, in the USA, pheasants were predominantly killed by foxes over their first winter, with 68% of 146 confirmed hen mortalities attributed to mammalian predators (especially red foxes) and 14% to raptors (Perkins et al. 1997).

Predation on incubating hens during the breeding season varies between sites. Draycott et al. (2008) reported 33/361 (9%) of radio-tagged hens predated on or off the nest during the breeding season at five UK sites. There was an extreme case of 80% of reared hens predated whilst nesting compared to 27% of wild-born hens at the same site (Hill and Robertson 1988a). Raptors again play a lesser role, at this time, accounting for 4.7% of predation of reared pheasants in Sweden (Brittas et al. 1992).

#### Factors that exacerbate predation levels of released birds

For ethical and financial reasons, game rearers use simple rearing environments, without adult conspecifics or predators and with monotonous food provided in excess. This results in reared pheasants lacking opportunities to learn predator identification and appropriate escape or avoidance responses (Dowell 1990). Rearing pens often lack perching opportunities, which may inhibit the learning of roosting behaviour and development of appropriate morphology (Hill and Robertson 1988b; Whiteside et al. 2016). Barren rearing environments likely restrict the development of flight muscles, by removing the incentive to fly, and thus, reared birds have poorer flight and escape capacity compared to wild pheasants (Robertson et al. 1993). We suspect that the barren environments provide the developing chicks with no cues as to suitable nesting sites, unlike wild-hatching chicks that can assess and perhaps imprint on their natal environment. A poor choice of nest site as adults may prove fatal to reared pheasants, with predation in the breeding season predominantly on poorly concealed nests (Chesness et al. 1968). Finally, release of large numbers of pheasants in itself may exacerbate predation, with high concentrations of birds attracting predators (Kenward et al. 1981; Robertson 1988; Roos et al. 2018).

#### Mitigation that may reduce predation

Predation rates are lower for wild-born pheasants compared to age-matched reared pheasants across multiple studies, implying that some critical aspects of early life differ between wild and reared birds (Brittas et al. 1992; Hill and Robertson 1988a; Krauss et al. 1987; Leif 1994; Musil and Connelly 2009). Some of these aspects can be reinstated by careful husbandry. The provision of raised perches in early life facilitates the development of elevated roosting at dusk and the associated morphology, resulting in enriched birds being more likely to roost off the ground and predated upon less (Santilli and Bagliacca 2017; Whiteside et al. 2016). Anti-predator training via the presentation of predator stimuli in early life influenced vigilance behaviour with captive-reared grey partridge, *Perdix perdix* (Beani and Dessì-Fulgheri 1998), and improved post-release survival of red-legged partridges *Alectoris rufa* and chuckar *Alectoris chuckar* (Gaudioso et al. 2011; Slaugh et al. 1992). Provision of a more complex and varied diet early in life can alter the foraging behaviour of reared pheasants, meaning that they exploit a wider natural diet, process prey more effectively, spend more time being vigilant and less time in exposed areas and subsequently survive better (Whiteside et al. 2015).

Post-release management can also reduce mortality, with high levels of predator control practised with this intention. Across seven sites in the UK, those with high levels of predator control had adult predation rates (30%) about half those at sites with low levels of predator control (60%) (Sage et al. 2018). However, we suspect that interpreting these results is confounded because all the low-predator-control sites were predominantly stocked with reared pheasants whereas two thirds of high-predator-control sites were unstocked and contained only wild pheasants. Therefore, fewer adults may be predated because they are wild-born, not because intense predator control was practised. Predator control has a somewhat more demonstrable effect on productivity, with a meta-analysis of 25 UK, European and US wild pheasant populations revealing a higher density of breeding hens and chicks fledged per square kilometre at sites with predator control, as well as stronger positive density-dependent productivity at sites with predator control (Sage et al. 2018). In the USA, predator control only increased pheasant numbers when practised at a very large scale (259- or 41-km^2^ plots). When control was practised over 10-km^2^ plots, larger than the vast majority of UK shooting estates, no effect was detected and the cost-efficiency of such measures was negligible (Chesness et al. 1968; Frey et al. 2003; Trautman et al. 1974). In Finland, a site with low natural fox populations hosted lower mortality rates of reared pheasants, matching levels comparable to those of wild birds living in a site of high predator abundance (Kallioniemi et al. 2015). A second post-release management technique involves supplementary feeding during spring which allows hen pheasants to spend less time foraging and more time close to protective cover, reducing exposure to predators (Hoodless et al. 2001).

### Disease and health

#### The effect of disease

Reared pheasants are commonly at risk from pathogens and parasites including *Heterakis gallinarum*, *Eucoleus contortus* and *Syngamus trachea* (Draycott and Parish 2000; Lund and Chute 1974; Millán et al. 2002). These pathogens and parasites can cause weight loss and significant mortality in heavily infected released pheasants (Gethings et al. 2016; Ruff 1999). Attributing death to disease, as distinct from, e.g. starvation, is difficult without a comprehensive post-mortem, but 3–19% of reared birds across multiple sites were found dead and unmarked by predators in their release pens (where supplementary food is abundant) (Robertson and Whelan 1987; Sodeikat et al. 1995; Turner 2007). Incidence of infection increases at high release concentrations (Draycott and Parish 2000), especially in release pens (Draycott et al. 2006). The effects of disease persist after pheasants leave the release pen. Approximately 20% of hunted wild pheasants (in Germany) exhibited protozoal cysts, hepatitis and/or enteritis (Curland et al. 2018). Predation rates were higher for helminth-infected pheasants, perhaps because of increased odour cues or reduced flight performance (Millán et al. 2002). Disease may prove especially damaging during the breeding season. Kidney damage, likely caused by the coronavirus, was a common clinical sign in hen pheasants during the breeding season (as high as 32% in 2011) (Draycott 2013). Worm burden was believed to be responsible for 10% of hen pheasant mortality during the breeding season in 2011 and 18% in 2012 (Draycott 2012; Draycott 2013). Breeding pheasants are frequently infested with ectoparasites such as ticks, *Ixodes ricinus*, which can cause exsanguination, reduced anti-predator vigilance and reduced visual perception (Hoodless et al. 2002).

#### Factors that exacerbate disease levels in released birds

Stressful conditions in early life may increase susceptibility to disease. Pheasants reared in barren conditions exhibited higher levels of tonic immobility, indicative of stress, than those reared in enhanced conditions (Hrabcakova et al. 2012). High-density releases may provoke competition and restrict access to resources such as water and shelter, which facilitates the effects of pheasant coronavirus and causes kidney failure precipitated later by chilling and water deprivation (Pennycott 2000). Furthermore, high rearing densities mean that disease can rapidly spread and can continue to influence disease risk post release. High pheasant densities also predict infection levels of *H. gallinarum* (Draycott and Parish 2000). The repeated use of rearing facilities can permit the build-up of infectious agents between cohorts of chicks (Gethings et al. 2015b).

#### Mitigation that may reduce disease

Management during rearing may serve to reduce stress, competition and resource limitation. This, in turn, can reduce susceptibility to disease. Provision of raised perches reduced aggression (Whiteside et al. 2016) and subsequently reduced feather damage (Santilli and Bagliacca 2017), likely reducing stress. Chicks hatched from the eggs of hens kept in captivity exhibited higher indicators of stress than those laid by wild parents (Santilli et al. 2004). Antioxidant supplementation, in the form of vitamin E, during rearing reduces future parasite loads and the oxidative stress associated with the maintenance of a high parasite load (Orledge et al. 2012b). Antibiotic and anthelminthic treatments are habitual in early life pheasant husbandry and commonly administered prophylactically (Broadfoot et al. 2017). Healthy flocks of pheasants medicated with a suite of antibiotics were ~ 18% heavier than control flocks and had lower death rates (Scott et al. 1954). Medication of pheasants with these antibiotics also improved feed conversion efficiency resulting in age- and dose-dependent increases in growth rates of 7–29% (Jukes et al. 1955). However, the longer-term effects of such elimination of gut microbial fauna in released birds and the broader risks of developing resistance, both for pheasant populations and the wider ecosystem (e.g. Radhouani et al. 2014), have not been explored.

Medication remains an effective management tool post release. Released hen pheasants provided with orally dosed antihelminthics had reduced worm burden and increased chick production twofold (Woodburn et al. 2002). Anthelminthic medication, administered via feed, reduced worm burdens and resulted in 25% more young being observed in areas with anthelminthic provision compared to control plots (Draycott et al. 2006). More male pheasants provided with acaricide necklaces (44%) acquired harems compared to controls (22%) (Hoodless et al. 2002) while treated females had higher survival and hatched 4.71 times more chicks per successful hen (Hoodless et al. 2003). In addition to the practical difficulties of medicating wild animals, long-term medication may not be practicable for harvested populations destined to enter the human food chain, and dosing schedules must comply with recommended withdrawal periods.

More broadly, disease risk can be reduced by decreasing stocking intensity, both spatially and temporally. Stocking density accounted for 47.2% of the variation in soil assays of *S. trachea* eggs, with more eggs found in pens with higher annual stocking density. Eggs remain in the soil, with pen age accounting for 38.4% of the variation in egg numbers, with older pens having higher egg numbers (Gethings et al. 2015b). Therefore, regular resting of pens between releases can reduce infection pressure in subsequent releases. For sites where it is not possible to move or rest pens, the highest concentration of eggs is found close to feeders (Gethings et al. 2015a), so moving feeders within and outside the pen is recommended to reduce transmission.

### Starvation

#### The effect of starvation

Two key factors can influence starvation: the food available in the environment and the ability for the pheasant to detect, handle, and digest that food (Thomas 1987). In many cases, starvation itself does not kill the individual, but hunger can make them engage in risky foraging or dispersal behaviour or depresses their immune system. However, in especially harsh conditions such as US winters, birds may die directly from starvation, with high mortality in snowy years (Errington 1939; Perkins et al. 1997). At the end of the shooting season, supplementary feed is often withdrawn (Draycott et al. 1998), and changes from spring to autumn sowing of cereals (O'Connor and Shrubb, 1986) have reduced the amount or winter stubble and wasted grain available to pheasants (Hoodless et al. 2001).

#### Factors that exacerbate starvation in released birds

Reared pheasants may face additional risk of starvation because they lack the characteristics necessary to acquire and process a nutritious diet. Reared pheasant chicks are provisioned with age-specific chick crumb, which is nutritionally balanced but monotonous in form and in excess, lacking diversity and naturalistic characteristics of wild chick diets. As such, reared game birds often differ from wild conspecifics in their digestive capability (Putaala and Hissa 1995) and their foraging and food-processing ability (Brittas et al. 1992; Sage and Robertson 2000). Furthermore, pheasant chicks reared in the absence of adults cannot acquire social information about feeding preferences, foraging sites or prey-processing methods. Therefore, many reared pheasants develop a high dependence on supplementary feed (Draycott et al. 1998), making the transition between supplementary fed diet and natural diet far harder (Draycott et al. 1998).

#### Mitigation that may reduce starvation

The effects of early-life diet can be profound for pheasants, persisting into adulthood. Small manipulations to the composition of pheasant diet during early development can influence tarsal size and symmetry (Ohlsson and Smith 2001), male sexual ornaments (Ohlsson et al. 2002), body condition (Sage et al. 2002), body size (Orledge et al., 2012aa) and primary feather development (Liukkonen-Anttila et al. 2002). The nutritional state of the mother laying the egg may also be critical, with hens fed standard rearing crumb producing offspring with poorer food-learning abilities than those of hens reared on a diet supplemented with fatty acids (Bagliacca et al. 2000).

Simple manipulations to the diet provided in the intensive rearing environment can have post-release behavioural and fitness consequences. Pheasants reared with access to a more naturalistic diet, with mealworms, mixed seeds and fruit supplementing commercial chick crumb, were more efficient at catching novel prey, had a more diverse post-release diet, a gut morphology to cope with high-energy foodstuffs and a more efficient foraging behaviour when in the wild (Whiteside et al. 2015). Five times more birds reared with the enhanced diet survived the first year than birds reared with control diet in 2012 and 2.4 times more treated birds survived in 2013.

The effects of diet manipulations later in life are more equivocal. The provision of supplementary food in later life did not increase the survival of hens on treatment plots (Hoodless et al. 1999). However, hens spent less time foraging for food and more time next to cover when provided with supplementary feed during the breeding season (Hoodless et al. 2001). This may be critical during incubation, when reared hens lost up to 40% of their body mass (Robertson et al. 1993), which may result in nest abandonment and death (Hoodless et al. 1999; Robertson 1997). This may explain why survival of reared birds during this period (4%) is lower than that of their wild conspecifics (40%) (Musil and Connelly 2009). Therefore, continued feeding of birds after the shooting season may benefit the population in terms of productivity and recruitment, even if it does not enhance individual survival.

### Dispersal

#### The effect of dispersal

In extended areas of dense pheasant release, dispersal from one estate may supplement stocks on neighbouring shoots, and therefore, dispersal itself may be inconsequential to the overall release/shot ratio. However, where shooting estates are not contiguous, dispersing birds likely leave managed areas and so enter areas without supplementary food, managed habitats or predator control where they are more likely to die. Additionally, from the perspective of the shoot owner, they cannot contribute to the harvest of the estate/farm, their intended consequence.

Data on dispersal by reared pheasants in the UK is limited. Movement prior to or during the shooting season may be revealed by recording birds shot beyond their release area. Across six sites over 3 years in the UK, 6% of released birds were reported shot on estates other than where they had been released (Turner 2007). This corresponds to our own data at three further sites where an average of 4.6% of 3352 tagged birds were reported shot on other estates. If these birds are shot in a ratio similar to birds on their natal estate, then we might expect that they represent ~ 40% of the birds that had actually reached the neighbouring estates, such that around 15% of released birds may have moved off their releasing estate during the shooting season. Pheasants that were deemed ‘bold’ in a battery of personality tasks did not disperse further than ‘shy’ birds (Madden and Whiteside 2014). Pheasants typically remain within a few kilometres of their release point. In the US, reared pheasants dispersed 1.6–3.2 km (Burger 1964; Harper et al. 1951; Kabat 1955; MacNamara and Kozicky 1949; Wilson et al. 1992). In the USA, pheasants that dispersed post-winter on average 3.2 km moved to areas with more open ground (Leif 2005). In the UK, pheasants moved an average of 30 m further from the release pen each day after release into the wild (Robertson 1986). Post-winter/pre-breeding movements are greater in first-year hens (309 m) compared to adult hens (196 m) and adult males (66 m) (Hill and Ridley 1987).

#### Factors that exacerbate dispersal in released birds

Pheasants released on areas with poor food and cover disperse more widely than those released in good pheasant habitat (Burger and Oldenburg 1972; Leopold et al. 1938; MacNamara and Kozicky 1949). Being reared under artificial conditions may cause different search and movement behaviour in released birds compared to their wild conspecifics. Wild pheasants may have greater dispersal because they try to find suitable habitat (Bagliacca et al. 2010). Wild pheasants in Italy also avoided areas of human activity compared to reared pheasants, possibly reducing risk of vehicle collisions or disturbance. In contrast, reared pheasants in the UK appear to prefer cover crops and sites with supplementary feeding (Turner 2007).

Pheasants are released at high densities, causing competition for food, water, roosting and nesting sites, which may stimulate dispersal for some individuals. The stocking density in a release pen did not influence movement of females in terms of distance moved, but males released from pens with high stocking densities had larger home ranges around the pen, suggesting that they were motivated to escape the area (Turner 2007). We also suspect that constant harassment during the shooting season in the form of disturbance by beaters and dogs may cause pheasants to disperse from release areas. The proportion of birds permanently dispersing from their release site decreased with releases later in the year (June–September) (Turner 2007).

#### Mitigation that may reduce dispersal

We believe that dispersal could be reduced by providing a post-release environment that reduces competition for food, water, shelter and refuge. This can be achieved by adding distribution devices that cannot be easily monopolised such as nipple drinkers and scatter feeders and have been shown to reduce conflicts in domestic hens (Gilani et al. 2013; Zimmerman et al. 2006). Likewise, the provision of numerous small supplementary feeding sites that cannot be monopolised may encourage retention of a higher density of territory-holding males during the breeding season (Hill and Robertson 1988b). Pheasants released on areas with sufficient food and cover disperse less widely than those released on poor pheasant habitat (Burger and Oldenburg 1972; MacNamara and Kozicky 1949). In the USA, a more diverse habitat allowed females to occupy smaller home ranges and hence disperse less (Schmitz and Clark 1999). Manipulation of artificial early-life conditions does not seem to affect dispersal propensity: rearing pheasants with foster hens did not alter their post-release dispersal compared to control birds (Ferretti et al. 2012), nor did rearing with naturalistic diet (Whiteside et al. 2015).

### Other causes of mortality: roadkill and agricultural operations

#### The effects of other causes

Losses to other causes of mortality are poorly understood. In the UK, ~ 7% of deaths of reared pheasants were attributed to factors other than predation and disease, with the majority (6%) being roadkill (Turner 2007). During the 1950s and 1960s, pheasants comprised ~ 7% of 3932 birds reported dead on roads (Dunthorn and Errington 1964; Hodson 1960; Hodson and Snow 1965)*.* An estimated three million pheasants are killed annually on UK roads (although there is no description of how this figure has been attained) (Anonymous 2015). This figure is not dissimilar to the extrapolated 2.1 million if 6% of the 35 million released pheasants are killed on roads. There are two distinct peaks in mortality occurring in late summer/early autumn immediately after birds have been released, and again in mid spring after the shooting season has ended and surviving birds likely start to search for new food supplies or establish territories (Madden and Perkins 2017). Leif (1994) noted that accidental deaths only occurred in reared pheasant populations and not among wild individuals that were studied. Mortality during the breeding season may also be due to destruction of nest sites with sitting hens. In the USA, ~ 30% of pheasant nests were destroyed by agricultural harvesting (Linder et al. 1960).

#### Mitigation that may reduce other causes of mortality

Given the paucity of data on the occurrence of other causes of mortality, suggestions for suitable mitigations are necessarily speculative. Later harvest periods may reduce destruction of nests. Continued provision of supplementary feeding into March–April may reduce dispersal by hungry birds and so reduce their exposure to traffic threats (Madden and Perkins 2017).

## Discussion

Pheasants that are reared and released in the UK suffer from high levels of loss. These losses can be highly variable across site and year, suggesting that they may be influenced by local management and ecological factors. Our review of the literature suggests that losses could be generally reduced by refinements in rearing practice and the management of the release habitat. While much effort is already expended on post-release management, often focussing on predator control or habitat creation, much less effort has been directed at pre-release management other than to maximise survival up to the point of release.

It is very difficult to predict exactly what effect any single modification is likely to have because single interventions may have multiple effects and the magnitude of these effects may be dependent on additional management or ecological conditions. In addition, there has been replicated testing of only two interventions: provision of perches in early life tested at two sites (Santilli and Bagliacca 2017; Whiteside et al. 2016) and diet enrichment tested in 2 years on the same site (Whiteside et al. 2015), limiting our confidence in the size of their effects. Consequently, we do not wish to specify the number of deaths that could be avoided. Additionally, we accept that some losses in any animal rearing system are inevitable, especially when the animals are free-living and can only be managed indirectly. For example, comparable figures for salmonid fish that are reared and released in running water (where they are unconstrained) for angling reveal that recapture rates are < 8% for fish released as fry/fingerlings and from 4 to 65% for fish stocked at a takeable size, suggesting pre-harvest mortality rates of 35–99% (Cresswell 1981). Finally, we acknowledge that a harvest rate of 100% is never possible because of human inaccuracy, inclement weather during the hunting season, etc. Therefore, even if natural mortality prior to the shooting season could be prevented, we do not expect that it would ever be feasible to only release as many pheasants as were intended to be shot. Instead, we are suggesting that any reduction in mortality brings benefits. For each 1% reduction in current mortality rates, around 350,000 fewer pheasants could be released while maintaining the numbers shot. Annually, this would bring an economic saving of ~ £4.5 million, remove ~ 385 tonnes of omnivorous biomass from the UK ecosystem and remove the need to rear 350,000 birds under artificial conditions.

### Changing release type

Perhaps the most intuitively obvious solution is to delay the release of birds until immediately (hours, days or few weeks) before hunters enter the area. This is practised in both Europe and the USA; however such ‘canned hunting’ is unpalatable to both those who are pro- and anti-shooting in the UK (Anonymous 2012). Furthermore, birds released immediately prior to shooting have not had the opportunity to learn the local environment and, thus, we believe that they will not fly in predictable directions when flushed (as do longer-term resident birds which can be driven towards release pens or roosting woods), making driven shooting less effective and possibly negating any benefits gained. In the USA where birds are often released straight from cages into the wild in the days prior to shooting, with no attention to feeding or predator management, returns indicate poor survival in the range 2–30% over a similar period of release (Burger 1964; Haensly et al. 1985; Hessler et al. 1970). In France, Mayot (2003) found that large open-topped pens as commonly used in the UK produced a return of shot birds 35% higher than the equivalent French method which entails the release of between 10 and 100 pheasants into small closed-topped pens for around a week, before being ‘trickled’ out in small batches. Consequently, we are not convinced that such a change in releasing practice will substantially reduce waste.

### Changing management actions

We have highlighted 14 methods which may be implemented by game managers during rearing and following release which we believe will likely reduce mortality of released pheasants (Table 1).

Two commonly used game management methods deployed following the release of pheasants (lethal predator control, supplementary feeding) have not yet definitively been shown to improve the survival of reared pheasants in the UK. Predator control does improve productivity of pheasants (and other species) and may improve the survival of adults outside the breeding season. However, this data is confounded by the origin of the pheasants, with more effort being put into predator control at sites where pheasants were not released but rather shooting depended on the wild population (Sage et al. 2018). Supplementary feeding did not directly increase the survival of released pheasants, but it did increase the numbers of pheasants in a fed area compared to controls (Draycott et al. 2005), as well as increasing the body mass and nesting efforts of birds in fed areas. Therefore, supplementary feeding may serve to raise the density of birds in an area through immigration, without improving their survival chances or the size of the wider population. Stronger evidence exists for the efficacy of post-release management enhancing survival in wild populations outside the UK. When predator control and supplementary feeding were applied simultaneously at a single site in Austria, they permitted an increased harvest of wild pheasants of 400–1350%, suggestive of improved survival up to the point of shooting (Draycott et al. 2002). The effects of such management are highly likely to be influenced by other variables in the local environment (e.g. natural predator abundance (Kallioniemi et al. 2015)) and the scale at which they occur (e.g. Frey et al. 2003). Further specific studies of predator control, similar to those conducted for other game bird species (Fletcher et al. 2010; Tapper et al. 1996), are required to test its efficacy at increasing survival of reared pheasants in the UK. Likewise, studies of supplementary feeding in the UK have been relatively small and localised, and we suggest a review of their efficacy and their extension to consider their effects in different conditions and locations. Such landscape-scale interventions (predator control and supplementary feeding) likely have unintended positive consequences for wildlife beyond game (Mustin et al. 2018; Roos et al. 2018). We suspect that both methods do improve the survival of released birds and effects are currently concealed because all game managers practise them over much of the UK with little opportunity for representative control sites to be assayed, but further, directed studies are required to confirm this.

In contrast, we found stronger evidence for the efficacy of some less common post-release interventions, specifically the use of anthelminthic and acaricide treatments, and a suite of pre-release management techniques. Of the interventions we surveyed, half of those which have demonstrable positive effects on survival are applied during the rearing period.

Early-life experiences can influence the development of behaviour, morphology, physiology and cognition (Buchanan et al. 2013; Lindström 1999; West-Eberhard 2003). Although reared pheasants survive the first 6–8 weeks of life far better than do wild chicks (Fig. 1), they die at much higher rates for the first 3 months following release. Therefore, the benefits of captive rearing in terms of boosting the population are rapidly lost. In contrast, wild birds that live to 8 weeks survive relatively well. This suggests that reared pheasants at 8 weeks old are lacking key behavioural, physiological or morphological attributes key to survival post-release. We believe that at least some of these attributes could be instilled in reared birds by simple changes to rearing practice. If captive rearing could promote the development of chicks so that survival rate between 2 and 5 months (currently 46.8%) matched that of wild-born chicks in that same period (86%), then some ~ 14–20 million fewer birds could be reared and released and yet still sustain the same harvest, potentially saving the industry > £180 million/year and reducing the biomass released into the wild by ~ 15,400 tonnes/year. Given the apparent wide range of benefits available, why are such practices not already implemented?

One explanation is that they add prohibitive costs to game rearing and keeping. We crudely estimated the economics of three interventions (pre-release diet enrichment and provision of perching, and post-release supplementary feeding beyond the shooting season; see ESM for details). They appear to be profitable and we suspect that some other cheap interventions such as moving feeding sites, enriching rearing pens, altering the diet of laying hens and oral medication could also be similarly profitable. A reduction in stocking density may even be profitable in itself. However, we suspect that some of the more intense interventions (medication—acaricide—by direct dosing, rearing with surrogate parents, large-scale habitat managements, predator control) may exhibit quite different balances.

A second explanation is that their deployment is retarded by inertia. Game management is typically a solitary job, with a single employee per site who generally works unsociable hours in isolated, rural locations (National Gamekeepers’ Organisation 2011). Consequently, there is limited opportunity or incentive for practitioners to share innovative methods. Shooting organisations with an interest in progressing and improving the industry, such as The British Association of Shooting and Conservation, the National Gamekeepers Organisation, the Game and Wildlife Conservation Trust or Countryside Alliance, all have effective means of communicating via press, game fairs, estate walks or workshops and should be encouraged to both disseminate and promote novel best practices. Such organisations and their members may also benefit, both in immediate financial ways and indirectly through improved public perception, from supporting further research of novel methods and should be facilitated to do so with links to the academic community.

### Important caveats

There is a serious risk that what we propose as interventions could be implemented simply to increase the efficiency of current game managers seeking to shoot a greater number of birds in total. This is not our intent. We hope that, by deploying these interventions, game managers can retain current harvests while reducing the numbers of birds that they rear and release. Alternatively, if regulatory limits on releasing pheasants are imposed, then these interventions may serve to ensure that returns are maximised after restricted releases.

A second risk arising from our interventions is that they have unintended consequences, both for the pheasants and the broader ecosystem, that have not been identified during their initial testing. Three speculative examples: encouraging young birds to forage for a diverse diet may make them harder to retain on an estate or impose increased predation on invertebrate populations in the release area; increasing survival when the birds are newly released may result in extended periods of high density which in turn may increase disease transfer or provoke dispersal into risky areas; and encouraging elevated roosting may reduce mammalian predation around the time of release, but these surviving pheasants may be predated by raptors, provoking conflicts of interest over the fates of these protected species. Clearly, future work should address possible impacts on a broader scale and over extended time. Nothing is known of the interaction between the interventions and the various strains of pheasants released. Existing intervention studies have been conducted on just one or a few sites; therefore, the interaction of early rearing interventions and the release environment is unknown. Further study of effects of early-life experiences is desirable, with particular focus on practices that are simple and cheap and can be applied on an industrial scale. It is also essential that such studies not only consider the intended consequences of the intervention but also account for possible unintended, detrimental consequences.

We believe that the common practice of post-release management in terms of predator control, habitat provision and supplementary feeding could be complemented by improved pre-release management practice. We believe (although we acknowledge that our understanding of the interventions is extremely limited) that those rearing pheasants for release for shooting should consider implementing a suite of interventions that are at present not commonly used in game farming and management. Breeders should source their eggs from unstressed, well-nourished adults. Pheasants should be reared under enriched and more natural conditions, ideally with the provision of elevated perching and a diverse diet from early in life. Pheasants should be released into an area that has had infectious hotspot locations such as feeder sites moved and where competition for resources is reduced. If such practices are implemented, then we believe that the natural mortality of released pheasants will be reduced and, thus, the number of pheasants released each year into the UK could also be reduced, bringing economic, environmental and ethical benefits to the shooting industry and a broader set of stakeholders.

## Electronic supplementary material

ESM 1 (DOCX 54 kb)
