# Supplementary material for: Why do many pheasants released in the UK die, and how can we best reduce their natural mortality?
Source: Eur J Wildl Res. 2018 Jun 22;64(4):40. doi: 10.1007/s10344-018-1199-5 (PMC7088407; doi:10.1007/s10344-018-1199-5)
Supplement: Supplementary file 1 — (DOCX 54 kb) [file 10344_2018_1199_MOESM1_ESM.docx]

**ELECTRONIC SUPPLEMENTARY MATERIAL**

1. *Differences in game management and shooting practice in the UK, US and Europe*

There is relatively little published work, especially over the past 25 years since the intensity of releasing has increased, on the ecology and behaviour of pheasants living in the UK. Therefore, we include data from the USA and Europe. However, it is important to note that the scale of shooting, the style of hunting and the game management techniques practiced in these other countries differ substantially from the UK. These differences mean that some direct comparisons between data from UK and non-UK based studies must be treated with caution.

In a single year (between 1998 and 2010), across Europe (excluding the UK) as a whole, around 1.8 million pheasants are shot, while in the USA, around 2.9 million pheasants are shot, compared to around 15 million shot in a single year in the UK (Mustin et al. 2011). Accounting for differences in land area reveals a density of shot pheasants in Europe and the USA of ~0.24 birds/km^2^ compared with ~61birds/km^2^ in the UK. This difference in the scale of pheasant shooting is partially attributable to the differences in hunting style: in Europe and the USA this typically takes the form of walked up or rough shooting, in which hunters and dogs walk through an area, shooting at birds as they encounter them, whereas in the UK it is typically driven shooting, in which groups of beaters and dogs corral pheasants from the surrounding area into a small wood or piece of cover from which they are driven to fly over a line of pre-positioned hunters (see Robertson (1997) for a fuller description of the hunting styles). In order to sustain such a high harvest levels, game management is typically much more intense in the UK with planting of woodland or cover crops to shelter and protect birds, provision of supplementary food (at least from release until the end of the shooting season), concerted attempts to reduce predation both by killing predators and deterring them, and high levels of stocking with hand-reared pheasants (e.g. see Robertson 1997). Whilst all these practices may be carried out in non-UK hunting areas, they typically occur at much lower levels.

1. *Methods of rearing and release of pheasants in the UK*

Pheasants bred for release are reared in large numbers under artificial conditions. Current rearing methods use mechanical incubators to hatch eggs and artificial brooders to intensively rear birds, without the need for parents. The system uses a relatively barren environment that is free from parasites and predators. Food, water and medication are provided adlib. Mechanical ‘bits’ are used to reduce the effects of aggression (Buner and Schaub 2008; Ferretti et al. 2012; Hill and Robertson 1988b). This process allows for game-rearers to produce high numbers of healthy birds whilst adhering to DEFRA codes of practice (DEFRA 2009). The early rearing environment can be tightly controlled and modified by the game manager.

Following rearing, pheasants undergo a ‘soft’ release into the wild aged 6-8 weeks old. Birds are initially placed into open-topped pens, enclosing an extensive area of woodland into which many hundreds, occasionally several thousands, of pheasants are released (GWCT 1991). The Game and Wildlife Conservation Trust recommends releases of <1000 pheasants per hectare of pen (Sage and Swan 2003). The primary function of the woodland release pen is to protect the young captive-reared birds from predators, in particular foxes, while they get used to roosting in trees or mature shrubs (GWCT 1991). During this period they also provide a controlled environment for feeding and, if required, disease management (GWCT 1988; GWCT 1991). Birds can disperse freely into the wild by flying out of the pen.

Game managers may assist free-living pheasants post-release in several ways. They commonly supply feed at set points before and throughout the shooting season (ending at the start of February), and some game managers continue feeding after this, until the breeding season (Draycott et al. 1998; Draycott et al. 2006; Hoodless et al. 2001). Managers can control predator numbers by lethal or non-lethal methods. Managers can also modify the landscape into which the pheasants are released, providing favourable habitats both during the shooting season to retain the birds in the shooting area, and for nesting or feeding areas to support birds after shooting has ceased. Such modifications may take the form of active planting or clearance, or deliberate neglect, or limits on application of pesticides.

Pheasant shooting requires a certain volume of birds to be shot in order to ensure profitability and economical sustainability. Therefore, we do not argue, in this paper, that the number of birds being shot *per se* is problematic, but rather that given such numbers need to be shot, what measures can be taken to ensure greater proportions of reared pheasants survive to contribute to the harvest, and thus by inference enable the rearing and release of fewer birds.

## *Why are interventions not implemented*

One explanation is that they are costly. We have crudely estimated the economic costs and benefits of three example interventions.

*Costs of enhanced feeding during rearing*

The cost of supplementing a standard commercial diet with seed and mealworms over the six week rearing period adds 12.3p (3.4%) *per capita* to the cost of a six-week old poult nominally costing £3.65 (SmithsGore and GWCT 2014). Delivering a diverse diet also incurs a small additional labour cost compared to traditional methods of feeding. However, this cost of £12.30/100 birds is recouped almost threefold if it means just one more of these 100 birds is shot and charged at the commercial rate of £35.12 (ex vat) (Savills and GWCT 2016).

*Costs of adding perching during rearing*

The provision of artificial perches in the form of plastic conduit piping costs £0.42 per meter. Current minimum welfare recommendations for intensively reared chickens suggest a perching distance of 0.15m per bird (Appleby 2003; CEC 1999). This adds 6p (1.9%) *per capita* to the cost of a six-week old poult for the first year of the study. Assuming that the perches persist for several years, this cost/bird is reduced. This maximum cost of £6/100 birds is again recouped almost six-fold if just one more of the 100 birds is shot.

*Costs of feeding beyond the shooting season*

Free-living pheasants require about 1.5tonnes/1000 birds/month (GWCT 2017a). This is a fixed cost for a shooting estate throughout the shooting season in order to hold birds in a particular area. Extending feeding beyond the shooting season inevitably adds costs, but such costs are likely to be markedly lower after the season has finished, with 40% of the original birds having been shot and further birds dying of other causes. Wheat costs are currently running at ~£135/tonne (Farmers Weekly 2017), such that each bird costs ~14p/month to feed so the cost of maintaining a bird up to the following August when feeding for the newly released cohort begins would cost 84p/surviving bird compared to a cost of £3.65 for a new poult purchased for release at the same time. Consequently, it is about four times cheaper to maintain and feed a bird after the shooting season has finished than buying a new one. In addition, a bird surviving through Spring may breed and so provide additional recruits to the population.

**ESM Table 1**

| 1. **Hand-reared Pheasants** | (Đorđević *et al*. 2010) | (Turner 2007) | (Hill and Robertson 1988a) | (Hoodless et al. 1999) | (Musil and Connelly 2009) | (Burger 1964; Hessler et al. 1970; Krauss et al. 1987; Trautman et al. 1974) |  |  | **Mean** | **Max** | **Min** | **Total pheasant (/100) alive at each stage** |
| --- | --- | --- | --- | --- | --- | --- | --- | --- | --- | --- | --- | --- |
| Location | Poland | UK | UK | UK | US | US |  |  |  |  |  |  |
| Early life Weeks 1-6 | 0.95 |  |  |  |  |  |  |  | 0.95 | 0.95 | 0.95 | 95 |
| Autumn survival (pre shooting) |  | 0.655 |  |  |  | 0.28 |  |  | 0.47 | 0.66 | 0.28 | 44.41 |
| Winter survival |  | 0.356 |  |  |  |  |  |  | 0.36 | 0.36 | 0.36 | 15.81 |
| Spring |  |  | 0.41 | 0.84 |  |  |  |  | 0.63 | 0.84 | 0.41 | 9.88 |
| Breeding season |  |  | 0.8 |  | 0.06 |  |  |  | 0.43 | 0.80 | 0.06 | 4.25 |
| 1. **Wild Pheasants** | (Clark et al. 2008) | | (Felley 1995) | (Wooley & Rybarczyk 1981) | (Dumke & Pils 1973) | (Snyder 1985) | (Hill & Robertson 1988a) | (Musil & Connelly 2009) |  |  |  |  |
| Location | Palo Alto | Kossuth | Idaho | Iowa | Wisconsin | Colorado | UK | US |  |  |  |  |
| Early life Weeks 1-6 | 0.46 | 0.37 | 0.25 |  |  |  |  |  | 0.36 | 0.46 | 0.25 | 36 |
| Autumn survival (pre shooting) | 0.86 | 0.86 |  |  |  |  |  |  | 0.86 | 0.86 | 0.86 | 30.96 |
| Winter survival | 0.66 | 0.61 |  |  |  |  | 0.65 |  | 0.64 | 0.66 | 0.61 | 19.81 |
| Spring | 0.79 | 0.84 | 0.67 | 0.544 | 0.71 | 0.674 |  |  | 0.70 | 0.84 | 0.54 | 13.96 |
| Breeding season |  |  | 0.79 | 0.697 | 0.856 | 0.833 |  | 0.4 | 0.72 | 0.86 | 0.4 | 9.99 |

Table ESM 1 Proportion of a) reared and b) wild-born pheasants surviving during one of five periods taken from a series of field studies in the UK, Europe and the US. These data were used to construct Figure 1 presenting the likely survival curve of populations of reared or wild-born pheasants.

**References for Data used in Table ESM1**

Clark WR, Bogenschutz TR, Tessin DH (2008) Sensitivity analyses of a population projection model of Ring-Necked Pheasants. The Journal of Wildlife Management 72:1605-1613

Burger GV (1964) Survival of ring-necked pheasants released on a Wisconsin shooting preserve. The Journal of Wildlife Management 28:711-721

Đorđević M, Pekeč S, Popović Z, Đorđević N (2010) Influence of dietary protein levels on production results and mortality in pheasants reared under controlled conditions. Acta Veterinaria 60:79-88

Dumke RT, Pils CM (1973) Mortality of radio-tagged pheasants on the Waterloo Wildlife Area. Wisconsin Department of Natural Resources Technical Bulletin 72. 52pp.

Felley, DL (1995) Recruitment and survival of ring-necked pheasants on the Nampa study area in southwestern Idaho. Paper 6895. <http://scholarworks.umt.edu/cgi/viewcontent.cgi?article=7930&context=etd> accessed 08 August 2017

Hessler E, Tester JR, Siniff DB, Nelson MM (1970) A biotelemetery study of survival of pen-reared pheasants released in selected habitats. The Journal of Wildlife Management 34:267-274

Hill D, Robertson P (1988a) Breeding success of wild and hand-reared ring-necked pheasants. The Journal of Wildlife Management 52:446-450 doi:10.2307/3801588

Hoodless AN, Draycott RAH, Ludiman MN, Robertson PA (1999) Effects of supplementary feeding on territoriality, breeding success and survival of pheasants. Journal of Applied Ecology 36:147-156

Krauss G, Graves H, Zervanos S (1987) Survival of wild and game-farm cock pheasants released in Pennsylvania. The Journal of Wildlife Management 51:555-559

Musil DD, Connelly JW (2009) Survival and reproduction of pen-reared vs translocated wild pheasants *Phasianus colchicus*. Wildlife Biology 15:80-88

Synder WD (1985) Survival of radio-marked hen ring-necked pheasants in Colorado. The Journal of Wildlife Management, 1044-1050.

Trautman CG, Fredrickson LF, Carter AV (1974) Relationship of red foxes and other predators to populations of ring-necked pheasants and other prey, South Dakota. In: Transactions of the North American Wildlife Conference, pp 241-252

Turner C (2007) The fate and management of pheasants (*Phasianus colchicus*) released in the UK. Imperial College

Wooley JB, Rybarczyk WB (1981) Pheasant population dynamics in southern Iowa. PR study completion report W-115-R, study, 2.
